# Reactive oxygen species act as signaling molecules to control root hair initiation and tip growth

**DOI:** 10.1111/nph.70306

**Published:** 2025-06-25

**Authors:** Megan E. Gerber, Maleana G. White, Gloria K. Muday

**Affiliations:** ^1^ Department of Biology Wake Forest University Winston‐Salem NC 27109 USA; ^2^ Center for Molecular Signaling Wake Forest University Winston‐Salem NC 27109 USA; ^3^ Molecular Genetics and Genomics Graduate Program Wake Forest University School of Medicine Winston‐Salem NC 27109 USA

**Keywords:** root hair, reactive oxygen species, respiratory burst oxidase homolog, class III peroxidase, hormone signaling, gene regulatory network

## Abstract

Root hairs (RHs) increase the surface area of roots, facilitating nutrient and water uptake and plant anchorage. RHs form from root epidermal cells and elongate by polar tip growth. Reactive oxygen species (ROS) have recently been implicated as important signals that drive RH formation and elongation using both genetic and imaging approaches. Localized changes in ROS levels in the RH tip are facilitated by hormone‐mediated changes in the synthesis and activity of respiratory burst oxidase homologs and class III peroxidases. These findings broaden our understanding of the mechanisms controlling polar tip growth in plants that drive RH formation, which can inform the breeding and engineering of plants that thrive under drought and nutrient stress.

## Español

Los pelos radiculares (PR) aumentan la superficie de las raíces, lo que facilita la absorción de nutrientes y agua, así como el anclaje de las plantas. Los PR se forman a partir de células epidérmicas radiculares y se elongan mediante el crecimiento polar del ápice radicular. Mediante métodos genéticos y de imagen, las especies reactivas de oxígeno (ERO) se han implicado recientemente como señales importantes que promueven la formación y elongación de los PR. Los cambios locales en los niveles de ERO en la punta de los PR se ven facilitados por cambios mediados por hormonas en la síntesis y actividad de homólogos de la oxidasa respiratoria y peroxidasas de clase III. Estos hallazgos amplían nuestra comprensión de los mecanismos que controlan el crecimiento polar del ápice radicular en plantas que promueven la formación de PR, lo que podría orientar el mejoramiento y la ingeniería genética de plantas que prosperan en condiciones de sequía y estrés nutricional.

## Português

Pelos radiculares (PRs) aumentam a área da superfície das raízes, facilitando a absorção de nutrients e água, bem como a ancoragem das plantas. PRs se formam a partir de células epidérmicas da raiz e se alongam pelo crecimento da ponta polar. Com o auxíilio de abordagem genéticas e de imagem, espécies reativas the oxigênio (EROs) foram recentemente implicadas como importantes sinalizadores que promovem a formação e o alongamento dos PRs. Alterações locais nos níveis de EROs na ponta dos PRs são facilitadas por alterações mediadas por homônios na síntese e na atividade de homólogos da oxidase respiratória e peroxidases de classe III. Essas descobertas ampliam nossa compreensão dos mecanismos que controlam o crescimento da ponta polar em plantas que promovem a formação dos PRs, o que pode informar o melhoramento e a engenharia genética de plantas que prosperam em condições de seca e estresse nutricional.

## 抽象的

根毛通过增加根的表面积以协助养分和水分的摄取以及植物的固定。根毛由根的表皮细胞形成，并通过极性顶端生长而伸长。近年来，借助遗传与成像手段，活性氧(ROS)被认为是驱动根毛形成和伸长的重要信号。激素介导的呼吸爆发氧化酶同源物和 III 类过氧化物酶合成和活性的变化促进了根毛顶端 ROS 水平的局部变化。这些发现拓宽了我们对于根毛极性顶端生长以及根毛形成机制的了解，并为培育与工程设计出能在干旱与营养胁迫下茁壮成长的植物提供了信息。

**Table:** 

	Contents	
	Summary	2042
I.	Introduction	2042
II.	Localized increases in ROS promote RH polar tip growth	2043
III.	Enzymatic regulation of ROS controls RH tip growth	2044
IV.	Hormones modulate ROS to control RH initiation and tip growth	2046
V.	Conclusions and future directions	2047
	Acknowledgements	2047
	References	2047

## I. Introduction

**Fig. 1:**
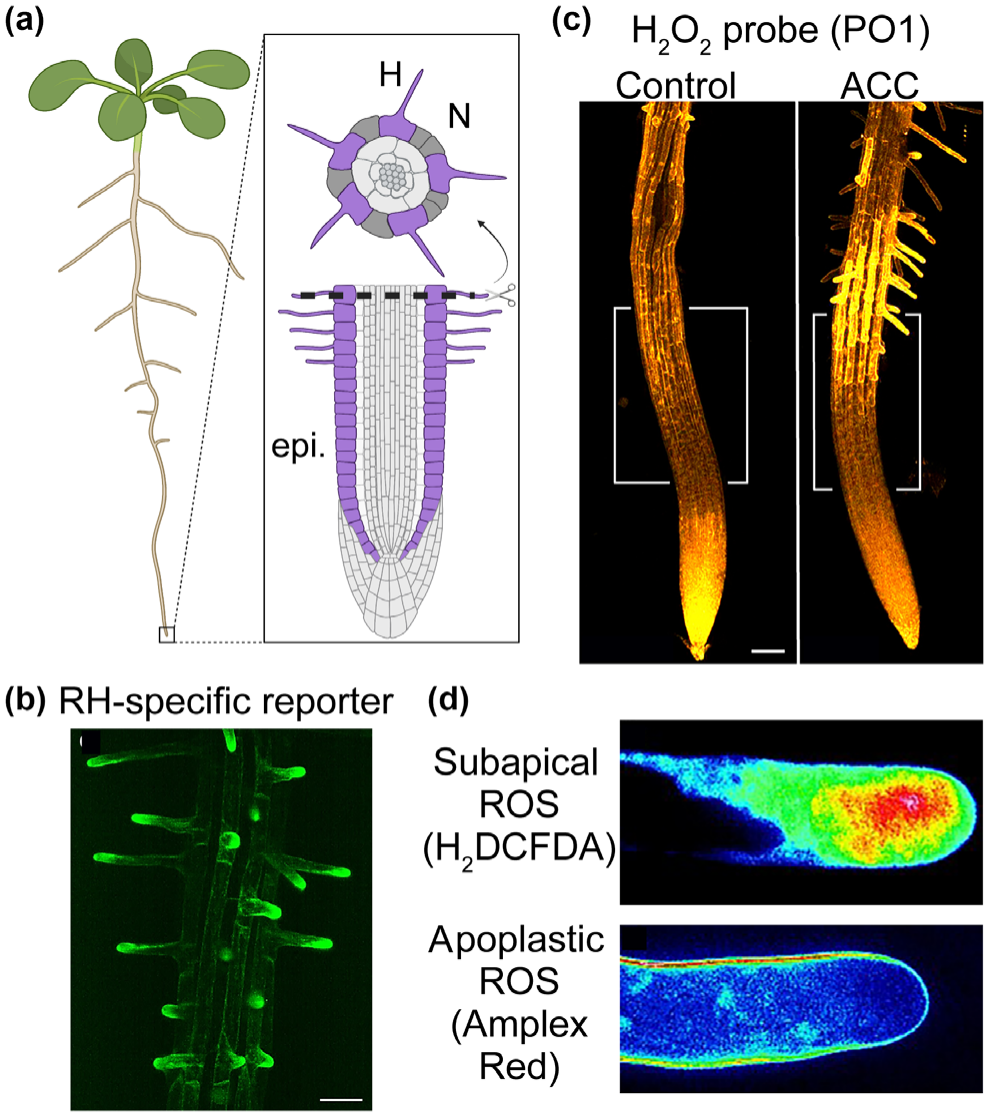
ROS accumulate in trichoblasts in distinct subcellular compartments to promote RH growth and development. (a) Schematic of an *Arabidopsis thaliana* seedling with the inset showing hair cells (H; trichoblasts) in purple and non‐hair cells (N; atrichoblasts) in dark gray in *cross‐* and *trans*‐sectional views. This figure was created in BioRender (BioRender.com/t2plfps) (Muday, G., 2025). (b) The ROS‐generating protein RBOHC/RHD2 is localized to the growing root hair tip as shown using *RHD2*::GFP‐RHD2 (reprinted from Ovečka *et al*., 2022). (c) The H_2_O_2_‐selective dye PO1 reveals localized increases in ROS in trichoblasts in response to ACC treatment (reprinted from Martin *et al*., 2022a). (d) RH cells stained with H_2_DCFDA and Amplex™ Red reveal subapical and apoplastic ROS, respectively (reprinted from Kuběnová *et al*., 2023). ACC, 1‐aminocyclopropane‐1‐carboxylic acid; GFP, green fluorescent protein; H_2_O_2_, hydrogen peroxide; H_2_DCFDA, 6‐chloromethyl‐2′,7′‐dichlorodihydrofluorescein diacetate; PO1, Peroxy Orange 1; RBOHC, respiratory burst oxidase homolog C; RHD2, root hair defective 2; RH, root hairs; ROS, reactive oxygen species.

Root hairs (RHs) are single‐cell projections that emerge from trichoblast cells in the epidermis, facilitating nutrient acquisition, soil anchorage, and root‐microbe interactions (Vissenberg *et al*., 2020). Root hairs emerge from epidermal cells in the differentiation zone near the root tip, with RH organization along the root developing in a species‐specific manner. In barley, a monocot, RHs form in alternating longitudinal patterns, while in other monocots RHs develop randomly (Marzec *et al*., 2015). In the model dicot plant, *Arabidopsis thaliana*, RHs form in a radial pattern of hair (H; trichoblast) separted by one or two non‐hair (N; atrichoblast) cells (Fig. 1a). *Root‐hair‐defective* (*rhd*) mutant screens in Arabidopsis revealed proteins required for RH initiation and tip growth, including RHD6 and RHD6‐LIKE 4 (RSL4) (Shibata & Sugimoto, 2019). Mutations in genes encoding these basic helix–loop–helix transcription factors (TFs) resulted in fewer initiation events and impaired elongation (Vijayakumar *et al*., 2016). Genetic screens also identified *root hair defective 2* (*rhd2*), which has impaired RH initiation and elongation (Schiefelbein & Somerville, 1990; Martin *et al*., 2022a). *RHD2* encodes RESPIRATORY BURST OXIDASE HOMOLOG C (RBOHC)/NADPH oxidase (NOX), an enzyme that catalyzes the transfer of electrons from NADPH to molecular oxygen, generating apoplastic superoxide, a reactive oxygen species (ROS) (Foreman *et al*., 2003; Chapman *et al*., 2019). ROS, including superoxide, hydrogen peroxide (H_2_O_2_), and hydroxyl radicals, can function as signals to control development (Martin *et al*., 2022b). The phenotypes of *rhd2* and localization of RHD2 to the apices of RHs (Fig. 1b) suggested a role for ROS in RH development. Transcriptome analyses and TF‐target assays determined that *RHD2* transcription is regulated by RHD6‐ and RSL4‐mediated gene regulatory networks (GRNs), supporting the involvement of RHD2 in RH formation (Vijayakumar *et al*., 2016; Feng *et al*., 2017).

Several other ROS producing enzymes, including RBOHs and multiple members of the plant‐specific class III peroxidase family, have been shown to modulate ROS levels in developing RHs. Mutations in these RH‐specific, ROS‐producing enzymes resulted in altered ROS accumulation and defects in RH development. Several of these enzymes function downstream of hormone‐mediated GRNs to locally induce ROS (Fig. 1c) (Mangano *et al*., 2017; Gayomba & Muday, 2020; Martin *et al*., 2022a; Lu *et al*., 2024). These findings revealed an important link between hormone‐mediated RH GRNs and ROS signaling in modulating RH formation and tip growth. We summarize recent insights into the ROS‐synthesizing enzymes and the hormone‐mediated GRNs that regulate these enzymes in Arabidopsis and crop species to control RH initiation and elongation.

## II. Localized increases in ROS promote root hair polar tip growth

Recent experiments have revealed localized ROS accumulation in trichoblasts during RH formation, which increases in response to hormonal and environmental conditions that drive RH proliferation (Martin *et al*., 2022b). Chemical probes, such as the general ROS sensor 6‐chloromethyl‐2′,7′‐dichlorodihydrofluorescein diacetate (H_2_DCFDA) and the H_2_O_2_‐selective dyes Peroxy Orange 1 (PO1; intracellular) and Amplex™ Red (extracellular), fluoresce upon oxidation to reveal higher H_2_O_2_ in trichoblasts than atrichoblasts (Fig. 1c,d) (Martin *et al*., 2022a; Kuběnová *et al*., 2023). RH tips have the highest H_2_DCFDA fluorescence in the subapical region (Fig. 1d), with decreased levels in *rhd2* compared to wild type (WT) (Foreman *et al*., 2003; Gayomba & Muday, 2020; Kuběnová *et al*., 2023). Amplex™ Red signal was highest in the apoplast of elongating WT RHs (Fig. 1d) and was abolished in *rhd2‐1* RH bulges (Kuběnová *et al*., 2023). These results are consistent with RBOHC‐dependent ROS accumulation promoting RH tip growth.

Perturbation of localized ROS levels in RHs alters their formation. Inhibition of RBOH activity by diphenylene iodonium blocked the synthesis of superoxide in the apoplast, decreasing RH elongation and mimicking the *rhd2* phenotype (Foreman *et al*., 2003). Similarly, treatment with the class III peroxidase (PER) inhibitor salicylhydroxamic acid resulted in stunted RH elongation (Pacheco *et al*., 2022). Conversely, treatments that increased ROS production increased RH length. For example, polyamines function as the substrates of polyamine oxidases, which synthesize H_2_O_2_. Trichoblast‐specific expression of a polyamine transporter resulted in increased polyamine accumulation and H_2_O_2_ in the RH apoplast, promoting RH elongation (Do *et al*., 2019). These results indicate that manipulation of ROS is sufficient to alter RH initiation and elongation.

ROS act as signaling molecules to regulate development through reactions with target proteins and other macromolecules to change their structure and activity. ROS produced in the apoplastic and subapical regions of RHs promote tip growth through similar mechanisms to those found in pollen tubes, including protein modifications, vesicle trafficking, cell wall remodeling, oscillatory tip‐focused gradients of Ca^2+^, and pH, which can modify ROS accumulation and vice versa (Lu *et al*., 2024; Zhang *et al*., 2025). The precise targets of ROS that modulate RH initiation and elongation are not all known, although H_2_O_2_‐mediated oxidative post‐translational modifications (oxPTMs) of cysteine thiols (‐SH) on target proteins have been identified in RHs (Lu *et al*., 2024; Mhamdi & Noctor, 2024; Zhang *et al*., 2025). In other plant tissues, these oxPTMs are found on TFs causing changes in transcriptional responses (Martin *et al*., 2022b; Mhamdi & Noctor, 2024). Accumulation of apoplastic hydroxyl radicals loosens the cell wall through glycosidic bond cleavage, while accumulation of apoplastic H_2_O_2_ promotes crosslinking of polysaccharides, increasing cell wall rigidity (Passardi *et al*., 2004). The identification of localized biochemical changes that drive RH formation is an important area of current research.

## III. Enzymatic regulation of ROS controls root hair tip growth

ROS homeostasis is maintained by a balance between enzymes that synthesize ROS and antioxidant systems that keep ROS from reaching damaging levels (Martin *et al*., 2022b). Studies in RHs have focused on the role of RBOHs and PERs as enzymes that synthesize and scavenge ROS to drive RH initiation and how specialized metabolites, like flavonoids, act as antioxidants to decrease ROS levels and limit RH formation (Daryanavard *et al*., 2023). In this section, we highlight recent insights into ROS‐producing enzymes controlling RH tip growth in Arabidopsis and crop species.

### Respiratory burst oxidase homologs

RBOH enzymes are highly regulated by hormones and their downstream signaling pathways, increasing ROS in both the apoplast and cytoplasm to drive RH initiation and tip growth. RBOH enzymes oxidize cytosolic NADPH, transferring electrons to oxygen to generate apoplastic superoxide, which is rapidly dismutated to H_2_O_2_, and can enter the cytoplasm through aquaporins (Chapman *et al*., 2019). RBOHs are integral membrane proteins with EF‐hand motifs that bind Ca^2+^ to regulate enzyme activity (Chapman *et al*., 2019). The RBOH gene family is large, with Arabidopsis, wheat (*Triticum aestivum*), rice (*Oryza sativa*), and maize (*Zea mays*) containing 10, 46, 9, and 15 *RBOH* genes, respectively. These genes have unique cell‐type expression patterns and distinct functions (Chapman *et al*., 2019).

In Arabidopsis, mutant analyses identified RBOHC, RBOHH, and RBOHJ as activators of RH initiation and/or tip growth, with RBOHC playing the largest role in these processes (Table 1). ROS synthesized by RBOHC activates Ca^2+^ channels, and Ca^2+^ activates RBOH enzyme activity, leading to synergistic activation of this response (Foreman *et al*., 2003). Hence, *rhd2* mutants fail to establish tip‐focused Ca^2+^ gradients that are required for RH tip growth, resulting in RH bulges rather than fully elongated RHs (Schiefelbein & Somerville, 1990; Foreman *et al*., 2003). RBOHC also plays a role in ethylene‐mediated RH initiation, with *rhd2* mutants initiating fewer RHs than WT when treated with the ethylene precursor 1‐aminocyclopropane‐1‐carboxylic acid (ACC) (Martin *et al*., 2022a). Additionally, the *rhd2* mutant has reduced actin dynamics in root epidermal cells, and proteomic analysis revealed an altered root proteome in this mutant (Takáč *et al*., 2024). Notably, *rhd2* had decreased abundance of an aquaporin that can move RBOH‐generated ROS from the apoplast into the cytoplasm. In addition to RBOHC, RBOHH and RBOHJ are active at the later stages of RH elongation, with *rbohh‐rbohj* double‐knockout mutants having shorter RHs and lower ROS levels than WT, although with a weaker phenotype than *rhd2* (Mangano *et al*., 2017). In pollen, RBOHH and RBOHJ are activated by Ca^2+^ to promote pollen tube elongation, though this mechanism remains untested in RHs (Kaya *et al*., 2014).

**Table 1:** Genes involved in ROS homeostasis that were demonstrated to function in RH growth and development.

Gene symbol (locus identifier)	Protein function in RHs	Regulation^1^	References
**Respiratory burst oxidase homologs**			
*AtRBOHC*/*RHD2* (At5g51060)	Promotes RH elongation and ethylene‐mediated RH initiation	RSL4; ROP2; Ca^2+^; CBL1‐CIPK26 (PTM); ↑ IAA, ↑ by ACC	Schiefelbein & Somerville (1990); Foreman *et al*. (2003); Lewis *et al*. (2013), Mangano *et al*. (2017); Martin *et al*. (2022a); Kuběnová *et al*. (2023); Lu *et al*. (2024)
*AtRBOHH* (At5g60010)	Promotes late‐stage RH elongation	Ca^2+^ ^2^; phosphorylation^2^	Kaya *et al*. (2014); Mangano *et al*. (2017); Bahmani *et al*. (2022)
*AtRBOHJ* (At3g45810)	Promotes late‐stage RH elongation	RSL4; Ca^2+^ ^2^; phosphorylation^2^	Kaya *et al*. (2014); Mangano *et al*. (2017); Bahmani *et al*. (2022)
*OsRBOHE*/ *OsRBOH3*/ *OsNOX3* (LOC_Os01g61880)	Promotes RH initiation and elongation; auxin‐mediated RH elongation in rice	Auxin signaling	Wang *et al*. (2018); Zhao *et al*. (2025)
*TaRBOH8‐A*/ *TaNOX3‐A* (TraesCS3A02G354200)	Promotes RH elongation	Multiple predicted regulators	Sharma *et al*. (2024); Tsang *et al*. (2024)
*ZmRBOHA*/*RTH5*/*GRMZM2G426953* (Zm00001d042961)	Promotes RH initiation and elongation	↑ by ABA, BR, and H_2_O_2_ treatments in leaves	Nestler *et al*. (2014)
**Class III peroxidases**			
*AtPER1*/At1g05240	Promotes RH elongation	RSL4	Mangano *et al*. (2017); Bahmani *et al*. (2022); Marzol *et al*. (2022)
*AtPER44*/At4g26010	Promotes RH elongation and ethylene‐mediated RH initiation	RSL4; ↑ by ACC; ↓ in microgravity	Kwon *et al*. (2015); Mangano *et al*. (2017); Bahmani *et al*. (2022); Marzol *et al*. (2022); Martin *et al*. (2022a)
*AtPER57*/At5g17820	Promotes RH elongation	↓ IAA, ↓ in microgravity	Lewis *et al*. (2013); Kwon *et al*. (2015)
*AtPER60* (*RHS18*)/ At5g22410	Inhibits RH elongation	RSL4; ↑ IAA; ↓ in microgravity	Won *et al*. (2009); Lewis *et al*. (2013); Kwon *et al*. (2015); Mangano *et al*. (2017)
*AtPER62*/At5g39580	Promotes RH elongation under cold stress	↑ by IAA; ↑ by BL	Goda *et al*. (2004); Pacheco *et al*. (2022)
*AtPER69*/At5g64100	Promotes RH elongation under cold stress	↑ by ACC; ↑ by IAA; ↑ by BL	Goda *et al*. (2004); Pacheco *et al*. (2022); Martin *et al*. (2022a)
*AtPER73* (*RHS19*)/ At5g67400	Promotes RH elongation	RSL4; ↑ by ACC; ↑ by IAA; ↓ in microgravity	Won *et al*. (2009); Lewis *et al*. (2013); Kwon *et al*. (2015); Mangano *et al*. (2017); Bahmani *et al*. (2022); Marzol *et al*. (2022)
*OsPER102*/ LOC_Os07g31610	Promotes RH elongation and inhibits RH thickening	Unknown	Moon *et al*. (2023)

ABA, abscisic acid; ACC, 1‐aminocyclopropane‐1‐carboxylate; *At*, *Arabidopsis thaliana*; BL, Brassinolide; IAA, indole 3‐acetic acid; BR, Brassinosteroid; CBL1‐CIPK26, Ca^2+^ sensor kinase complex; NOX, NADPH Oxidase; *Os*, *Oryza sativa*; PER, class III peroxidase; RBOH, respiratory burst oxidase homolog; RHD2, Root Hair Deficient 2; RHS, Root Hair Specific; RTH5, Roothairless5; *Ta*, *Triticum aestivum*; *Zm*, *Zea mays*.

^1^ Unless otherwise noted, molecular factors positively regulate RBOHs, and post‐translational modifications (PTMs) are kinase‐mediated phosphorylations. Up and down arrows refer to transcript abundance.

^2^ These findings were demonstrated only in pollen tube tips.

*RBOH* mutations are also linked to RH initiation and elongation defects in crop species (Table 1). The maize *roothairless 5* (*Zmrth5*) mutant has a defect in *ZmRBOHA*, forming fewer and shorter RHs with less ROS accumulation in RH bulges than WT, as monitored by H_2_DCFDA fluorescence (Nestler *et al*., 2014). In rice, a mutation in *OsRBOHE/OsNOX3*, an ortholog of *AtRBOHC* and *ZmRTH5*, had fewer and shorter RHs than WT and reduced H_2_DCFDA fluorescence (Wang *et al*., 2018; Zhao *et al*., 2025). In wheat, the *short root hair 1* (*srh1*) mutant was mapped to the *TaNOX3‐A* gene and had a significant reduction in H_2_DCFDA signal intensity in RH bulges compared to WT (Tsang *et al*., 2024). Transcriptome analysis of *Tasrh1* identified altered abundance of multiple TFs predicted to function in RH elongation consistent with a ROS‐regulated GRN. These results provide genetic evidence for RBOHs controlling RH initiation and elongation in Arabidopsis and crop species.

### Class III peroxidases

The plant‐specific PER family also regulates apoplastic ROS levels and RH development (Passardi *et al*., 2004). PERs (also abbreviated as PRXs, PODs, and POXs) are secreted, heme‐containing enzymes with three distinct catalytic cycles that consume H_2_O_2_ and superoxide or produce hydroxyl radicals, hydroperoxyl radicals, or superoxide (Passardi *et al*., 2004). PERs are part of large gene families, with Arabidopsis, wheat, maize, and rice genomes containing 73, 374, 119, and 138 *PERs*, respectively. The peroxidative cycle metabolizes H_2_O_2_ to facilitate cell wall lignification (Passardi *et al*., 2004). Paradoxically, PERs also promote cell wall loosening via the hydroxylic cycle, producing hydroxyl radicals that react with polysaccharides, facilitating cell expansion (Passardi *et al*., 2004).

Multiple PERs function in RH development and stress response in Arabidopsis (Table 1). The mutants *per1*, *per44*, and *per73* have mild reductions in RH length and less ROS accumulation in RHs compared to WT, which are accentuated in a triple mutant (Mangano *et al*., 2017; Bahmani *et al*., 2022; Martin *et al*., 2022a). Under microgravity, PER44, PER57, and PER73 have decreased expression resulting in shorter RHs (Kwon *et al*., 2015). During cold stress, PER62 and PER69 increased apoplastic ROS and decreased cytoplasmic ROS levels to drive RH elongation (Pacheco *et al*., 2022). Interestingly, overexpression of PER60, also named ROOT HAIR SPECIFIC 18 (RHS18), reduced RH tip growth compared to WT (Won *et al*., 2009). Unique among the functionally tested PERs, RHS18 acted as a negative regulator of RH elongation and might be a control mechanism to balance ROS production in elongating RHs.

In crop species, transcriptional analyses of RH‐defective mutants identified multiple PERs as potential regulators of RH elongation. In wheat, transcriptome analysis of two long‐RH genotypes and two short‐RH genotypes revealed increased abundance of *TaPER12*, *TaPER47*, and *TaPER56* in the long‐RH genotypes (Zeng *et al*., 2024). In maize, *ZmPER1*, whose encoded protein is a homolog of AtPER62, was upregulated in epidermal cells during cold stress‐induced RH initiation (Zhou *et al*., 2024). In rice, *Osper102* knockout mutants exhibited shorter RHs with defects in cytoplasmic streaming, although H_2_DCFDA staining did not reveal defects in ROS accumulation when compared to WT (Moon *et al*., 2023). These findings suggest that PERs play important roles in maintaining ROS gradients required for RH elongation through diverse functions and expression patterns.

## IV. Hormones modulate ROS to control root hair initiation and tip growth

Phytohormones can regulate ROS accumulation, activating GRNs that synthesize proteins to alter ROS accumulation in trichoblasts. The hormones auxin, ethylene, cytokinin, strigolactone, and jasmonic acid positively regulate RH formation and tip growth (Vissenberg *et al*., 2020). Treatments that elevated auxin and ethylene signaling in Arabidopsis increased H_2_O_2_ levels in trichoblasts, including RHs, as visualized with PO1 and H_2_DCFDA (Gayomba & Muday, 2020; Martin *et al*., 2022a; Kuběnová *et al*., 2023). Mutants with constitutive ethylene signaling had increased trichoblast H_2_O_2_ levels, RH initiation, and elongation, while mutants with reduced ethylene signaling had decreased ROS levels and fewer RHs (Martin *et al*., 2022a).

**Fig. 2:**
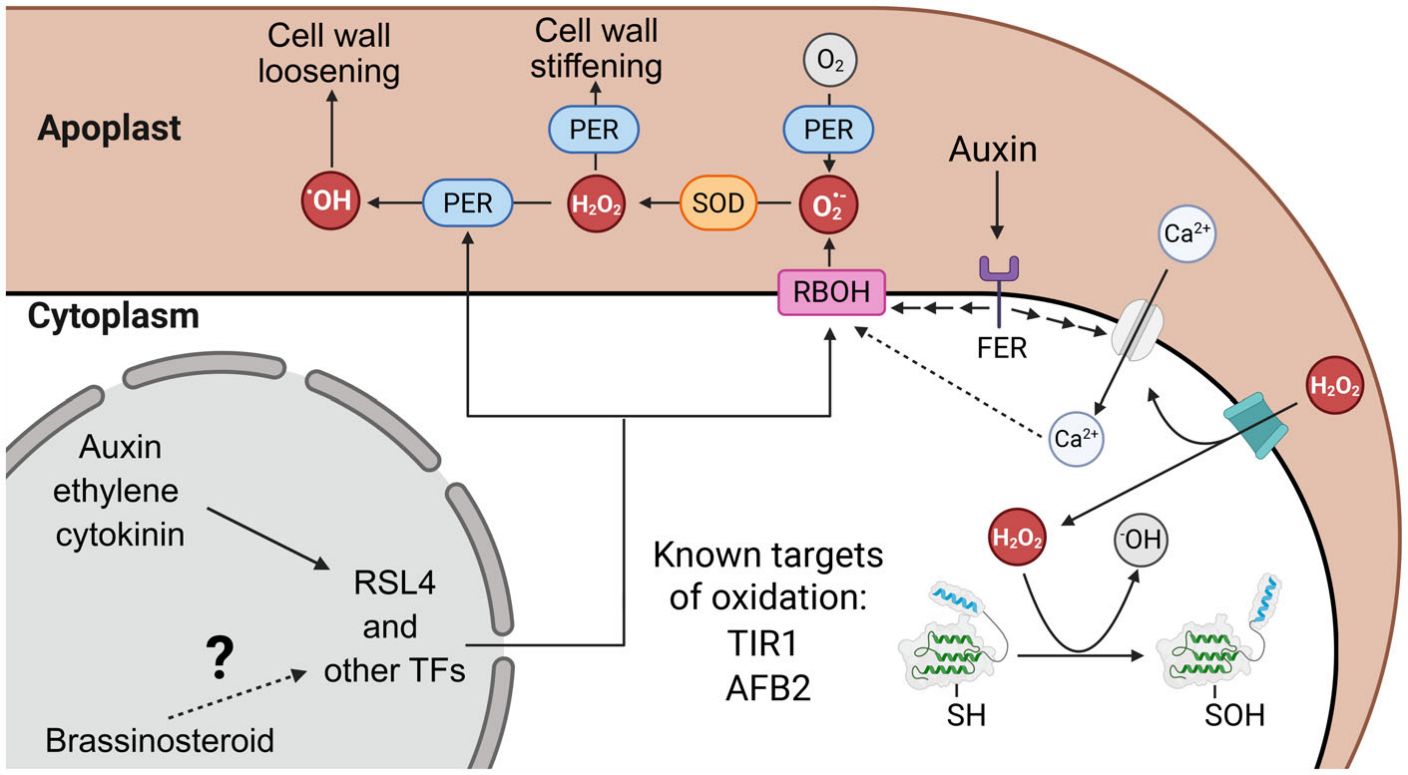
Model illustrating how hormone‐mediated ROS controls RH tip growth in *Arabidopsis thaliana*. Auxin, ethylene, and cytokinin regulate the gene encoding RSL4, which transcriptionally activates several ROS‐producing enzymes including respiratory burst oxidase homologs (RBOHs) and class III peroxidases (PERs). Brassinosteroids have been shown to increase ROS and root hair proliferation, but the mechanism is still unknown. RBOHs produce superoxide (O_2_^ᐧ−^) in the apoplast by reducing cytosolic NADPH. Superoxide can be converted into H_2_O_2_ spontaneously or enzymatically by SUPEROXIDE DISMUTASE (SOD). H_2_O_2_ is metabolized by PERs to promote cell wall stiffening. Alternatively, PERs convert H_2_O_2_ to hydroxyl radicals (ᐧOH) to facilitate cell wall loosening. PERs can also oxidize molecular oxygen to create superoxide. RBOHs can be activated by cytosolic calcium. RBOHC is activated by auxin signaling in the apoplast via the FERONIA (FER) receptor‐like kinase. FER activates RBOHC by multiple trafficking and/or signaling pathways (indicated by arrows). FER also controls calcium entry into RHs through MILDEW RESISTANCE LOCUS‐O 15 (MLO15) and other calcium channels (white), but the mechanism for FER regulation of MLO15 is still unknown. H_2_O_2_ enters the cytoplasm through aquaporins (blue) where it may activate calcium channels (white) or induce oxPTMs on target proteins, which activate secondary signaling pathways. Created in BioRender. Muday, G. (2025) https://BioRender.com/z1edjoq. Arrows through enzymes indicate enzyme‐catalyzed reactions. Dotted arrows indicate unconfirmed in root hairs or unknown. AFB2, Auxin Signaling F‐Box 2; H_2_O_2_, hydrogen peroxide; ^−^OH, hydroxide anion; RH, root hairs; ROS, reactive oxygen species; RSL4, RHD6‐LIKE 4; TF, transcription factor; TIR1, transport inhibitor response 1.

Hormones increase ROS synthesis by activating TFs that control the abundance of *RBOH*s and *PER* transcripts (Fig. 2). ETHYLENE INSENSITIVE 3 and RHD6 work together to induce transcription of *RSL4*, and several auxin response factors bind to the *RSL4* promoter to induce its expression (Feng *et al*., 2017; Mangano *et al*., 2017). Cytokinin also increases *RSL4* transcript abundance (Takatsuka *et al*., 2023). RSL4 binds directly to the promoters of genes encoding ROS‐producing enzymes including RBOHC/H and PER1/44/60/73 (Table 1). Consistent with PERs being downstream of the RSL4 GRN, PER activity decreased by roughly 60% in the *rsl4* mutant compared to WT (Mangano *et al*., 2017). Brassinosteroid also regulates the transcript abundance of *PERs* known to function in RH development. *PER62* and *PER69* were upregulated in response to both auxin and brassinosteroid treatments in a whole‐seedling, transcriptome dataset (Goda *et al*., 2004).

Synergistic interactions between hormones and ROS regulate RH development. Auxin uses the plasma‐membrane‐localized FERONIA receptor‐like kinase to activate RBOHC proteins. Recently, FERONIA has been shown to regulate Ca^2+^ oscillations in RHs through MILDEW RESISTANCE LOCUS‐O proteins, further inducing ROS production through Ca^2+^ (Ogawa *et al*., 2025). FERONIA‐dependent ROS accumulation results in oxPTMs on TRANSPORT INHIBITOR RESPONSE 1 and AUXIN SIGNALING F‐BOX 2, components of the canonical auxin signaling pathway (Lu *et al*., 2024). This results in their translocation from the cytoplasm to the nucleus to activate auxin signaling. Hormones also promote the production of specialized metabolites with antioxidant activity to maintain the balance of ROS in roots (Lewis *et al*., 2011). Mutants with defects in genes encoding flavonol biosynthetic enzymes have elevated ROS in epidermal cells and increased RH numbers, which can be genetically and chemically complemented, reversing the RH phenotype (Gayomba & Muday, 2020). Imaging revealed lower levels of flavonols and flavonol biosynthetic enzymes in epidermal cells, allowing elevated ROS that drives RH initiation. By contrast, cortical cells have higher flavonol abundance and lower ROS to prevent oxidative damage (Gayomba & Muday, 2020). Consistent with a protective role of flavonols in cortical cells, auxin and ethylene induced flavonol synthesis in cortical cells (Lewis *et al*., 2011). These findings reveal the role of hormone signaling in controlling ROS and antioxidant accumulation needed to fine‐tune RH growth and development.

## V. Conclusions and future directions

RHs are powerful models for studying polar tip growth in plants. Recent studies in Arabidopsis and crop species have revealed the enzymes that synthesize ROS in RHs to drive their formation and elongation as well as the GRNs that regulate these enzymes. Nevertheless, further work is needed to uncover how hormones control ROS synthesis outside of RSL4‐dependent GRNs. Additionally, the biochemical mechanisms by which ROS drive RH growth and development are an active area of study. Hormone treatment paired with proteomics analysis that identifies reversibly oxidized proteins, for example, could reveal the downstream targets of hormone‐induced ROS in RHs. Additional work is also needed to elucidate the role of hormone crosstalk in controlling ROS accumulation in trichoblasts through oxidant and antioxidant mechanisms. Finally, dissecting the role of the large PER family requires further investigation into the functionality of these proteins as ROS synthesizers and scavengers. Taken together, the work highlighted in this review broadens our understanding of RH growth and development and provides an avenue for exploring similar mechanisms in other tip‐growing cells.

## Competing interests

None declared.

## Disclaimer

The New Phytologist Foundation remains neutral with regard to jurisdictional claims in maps and in any institutional affiliations.
